# A110 FEASIBILITY OF EUS-GUIDED PORTAL PRESSURE GRADIENT MEASUREMENT WITHOUT DEEP SEDATION: A PATH TO ACCURATE PORTAL PRESSURE DETERMINATION

**DOI:** 10.1093/jcag/gwab049.109

**Published:** 2022-02-21

**Authors:** X Zhao, Y Chen, M Deschenes, P Wong, G Sebastiani, T Chen, A Benmassaoud

**Affiliations:** 1 Gastroenterology and Hepatology, McGill University Health Centre, Montreal, QC, Canada; 2 Divison of Gastroenterology and Hepatology, McGill University Health Centre, Outremont, QC, Canada; 3 Hepatology, MUHC, Montreal, QC, Canada; 4 Gastroenterology, McGill University, Brossard, QC, Canada; 5 Royal Victoria Hospital, McGill University Health Center, Montreal, QC, Canada; 6 McGill University Health Centre, Montreal, QC, Canada; 7 McGill University Health Center, Montreal, QC, Canada

## Abstract

**Aims:**

Hepatic venous pressure gradient (HVPG) is the gold standard for the diagnosis and staging of portal hypertension. Previous studies have shown that it underestimates pre-sinusoidal portal hypertension. Endoscopic ultrasound-guided portal pressure gradient (EUS-PPG) can safely measure direct portal vein pressure (PVP) and bridge this diagnostic gap. However, EUS-PPG has so far been performed with patients under deep sedation which can alter HVPG measurement and lead to misclassification of portal hypertension. Ketamine, a conscious sedation agent, has minimal effect on portal hemodynamics. We present our center’s experience with EUS-PPG under ketamine and low dose midazolam.

**Methods:**

We retrospectively reviewed consecutive patients undergoing EUS-PPG with conscious sedation using ketamine and low-dose midazolam (<0.02 mg/kg) at the McGill University Health Centre from February to May 2021. Patients were placed in the left lateral position. Hepatic vein and portal vein were located through EUS via a trans-gastric/hepatic approach. A through the scope 25-gauge needle attached to a manometer was advanced through the liver to measure hepatic vein pressure (HVP) and portal vein pressure (PVP). Three measurements were sampled per vessel and mean pressure differences calculated to obtain PPG. PPG was considered reliable if the differences between the values was no more than 1mmHg.

**Results:**

Three patients underwent EUS-PPG for evaluation of pre-sinusoidal portal hypertension. Cirrhosis was excluded in all patients based on recent liver biopsy or transient elastography. The first patient is a 69-year-old man with splenomegaly and recanalization of the paraumbilical vein on imaging. He received 80 mg of ketamine and no midazolam. HVPG measured was 1 mmHg. EUS-PPG was successful with PPG of 6 mmHg (HVP= 7 mmHg, and PVP= 13 mmHg). The second patient is a 42-year-old woman with previous sleeve gastrectomy and known with portal cavernoma. She received 120 mg of ketamine and 1 mg of midazolam. EUS-PPG was technically difficult due to respiratory movements. The measurement was not considered reliable as differences in PPG were as high as 2mmHg. In this case, PPG was 0mmHg (HVP= 15mmHg, and PVP= 15mmHg). The third patient, a 74-year-old man with hepatic steatosis and splenomegaly received 70 mg of ketamine and 1 mg of midazolam. EUS-PPG was successful (HVP= 3mmHg, PVP= 3mmHg) yielding a PPG 0mmHg. All patients tolerated the procedure well with no procedural or sedation-related complications

**Conclusions:**

Our early experience suggests that EUS-PPG can be successfully and safely performed in patients under conscious sedation with ketamine and low-dose midazolam. This combination may avoid deep sedation with high dose midazolam or propofol which are known to alter accuracy of HVPG.

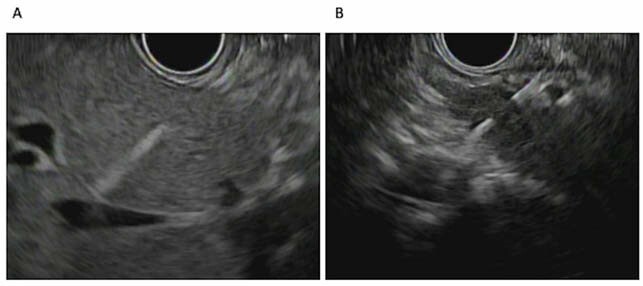

Measurement of HVP (left) and PVP (right)

**Funding Agencies:**

None

